# Understanding knowledge, attitudes and behaviours related to dietary sodium intake in a multi-ethnic population in Singapore

**DOI:** 10.1017/S1368980023002422

**Published:** 2023-12

**Authors:** Cindy Mei Jun Chan, Borame Sue Lee Dickens, Mary Foong-Fong Chong

**Affiliations:** 1 Saw Swee Hock School of Public Health, National University of Singapore and National University Health System, 117549 Singapore; 2 Singapore Institute for Clinical Sciences, Agency for Science, Technology and Research, Singapore

**Keywords:** Salt reduction, Dietary sodium, Knowledge, Attitudes, Practices

## Abstract

**Objective:**

This study aimed to fill the current gap in the understanding of the knowledge, attitudes and behaviours (KAB) related to dietary Na among adult residents in Singapore.

**Design:**

A cross-sectional online survey was conducted between October and December 2020 on 955 participants selected through random sampling.

**Setting:**

The survey was conducted in Singapore.

**Participants:**

Participants were recruited from the Singapore Population Health Study Online Panel.

**Results:**

Participants’ mean age was 46·6 ± 14·1 years old and 58 % of them were females. Most of the participants were Chinese (82·1 %), 10·5 % were Indian and 4·5 % were Malay. Findings from the weighted data showed that most participants were aware of the health impact of high Na consumption. However, many participants were unaware of the recommended intake for salt (68%) and Na (83%), had misconceptions, and were unable to correctly use food labels to assess NA content (69%). Findings also alluded to the presence of knowledge gaps in the sources of Na in their diet. While 59 % of the participants reported to be limiting their consumption of Na, many reported facing barriers such as not knowing how to limit their Na intake. Participants also felt that there were limited options for low-Na foods when eating out and were lacking awareness of low-Na products.

**Conclusions:**

Findings highlighted substantial gaps in participants’ knowledge and skills in managing their Na consumption. This suggests the need for more public education and improvements in the food environment.

Extensive research has established that high Na intake (largely via consumption of salt) is associated with high blood pressure, a key risk factor for CVD^(1)^. There is also increasing evidence that high Na intake is associated with many other age-related diseases such as kidney diseases, osteoporosis and stomach cancers^(2–5)^. More recently, it has been suggested that the observed increasing trends in deaths and disability are attributed in part to high Na intake, and that this trend would continue to increase due to the growing ageing population^(6,7)^. It is, therefore, pertinent to limit the Na intake of individuals. The WHO recommends that adults should consume less than 2 g of Na (equivalent to 5 g of salt) per day^(8)^. However, the salt consumption in many countries has remained consistently higher than the WHO’s recommendations^(9)^. Similarly in Singapore, the population has been consuming more salt than they should over the past decade. The average Na intake reported in the 2010 National Nutrition Survey was 8·3 g/d and further increased to 9 g/d in 2018^(10,11)^. Intakes at both time points were higher than the recommended intake of 5 g/d.

While there has been a focus on improving diets in Singapore in the recent years, the aims of these programmes (e.g. Healthier Choice Symbol programme^(12,13)^, Healthier Dining Programme^(14)^ and Healthier Ingredient Schemes^(15)^) were mostly on providing consumers foods or meals that are lower in calorie and sugar and higher in wholegrains, but less on Na reduction. As such, the Singapore Ministry of Health has recently announced the introduction of a multifaceted strategy to reduce consumption and increase awareness on dietary Na ^(16)^. Recent work by Tan *et al.* ^(17)^, which modelled the effects of four salt-reduction interventions on acute myocardial infarction and stroke incidence, has highlighted the importance of salt-reduction initiatives in the Singapore population. The authors proposed that a reduction of at least 2 g/d of salt consumption will be effective in averting a substantial number of incident cases of acute myocardial infarction and stroke and disability-adjusted life years over 30 years^(16)^.

Understanding the knowledge, attitudes and behaviours (KAB) of the population towards dietary Na^(18)^ can inform the development of effective salt-reduction interventions. For example, research assessing the KAB related to dietary Na conducted in high-income Western populations have identified sub-groups with poor knowledge of Na and salt intake recommendations, misconceptions about salt, lack of usage of food labels for salt reduction and lack of willingness to use low-salt products^(19–21)^. Such information has been beneficial in informing policy decisions and programs, as well as product reformulation and packaging by food industries. However, these findings may have limited generalisability to the multi-ethnic Asian population in Singapore due to differences in the cultural context and dietary habits. For example, while Na in the Western diet come mostly from processed foods, bread, cereal products and table salt, Na in Asian cuisine comes largely from a combination of sauces (soya sauce, fish sauce), seasoning (stock cubes and sodium glutamate) and table salt^(12,13)^.

To our knowledge, the understanding of KAB related to dietary Na in the Singapore population is lacking. Such data are valuable as a form of needs assessment prior to the implementation of Na-reduction initiatives and provides the opportunity to evaluate the impact and effectiveness of these interventions. This study thus aims to fill the current gap in the understanding of the KAB related to dietary Na among Singapore residents.

## Methods

### Study design and participants

A cross-sectional online survey was rolled out in Singapore between October and December 2020. Participants were recruited from the Singapore Population Health Study Online Panel maintained by the Saw Swee Hock School of Public Health, National University of Singapore. The online panel included individuals who are Singapore citizens or permanent residents, aged 21 years and above, frequent user of the internet, has a personal email account and able to read English as surveys were only conducted in English. Only one participant was invited from each household from the Singapore Population Health Study Online Panel, and they were randomly selected. After excluding multiple participants per household, invitations to participate in this online survey were sent to 1240 participants via email or text message. Those who consented to participate were provided a link to complete a questionnaire administered on REDCap, a secure web-based software platform designed to support data capture for research studies^(22,23)^, hosted at the National University of Singapore. The link to the questionnaire can be assessed by any forms of devices including desktop, laptop, tablet and mobile phone. Participants took an average of 15·5 min to complete the questionnaire and received $10 cash reimbursement upon completion of the questionnaire.

### Survey questionnaire

While a few dietary Na KAB studies in Asian countries, such as China, India and Hong Kong, have used questionnaires adapted for the Asian context^(20,21,24,25)^, we found that these questionnaires tended to focus on salt intake and did not enquire about other Na sources, such as sauces and seasonings. Thus, a questionnaire consisting of fifty-nine items was developed to assess Singapore resident’s knowledge, attitudes and behaviours towards their intakes of dietary Na. The KAB indicators consolidated by Bhana *et al.* ^(19)^ were used as a guide in the questionnaire development (See Appendix S1). The questions were adapted from existing KAB questionnaires administered in the USA^(26–28)^, United Kingdom^(29)^, Australia^(30)^, China^(24)^, Hong Kong^(25)^ and India^(21)^ and modified for relevance to the local context and ethnic cooking methods. The questionnaire was pilot tested for face validity among internal staff prior administration to the participants.

#### Knowledge

Sixteen items assessed participants’ declarative and procedural knowledge, which refers to the ‘awareness of things and processes’ and the ‘awareness of how to do things’, respectively^(19)^. Questions related to declarative knowledge included the relationship between salt and Na, dietary recommendations for salt and Na, and misconceptions about dietary Na intake. To assess procedural knowledge, two questions asked participants to compare the Na content of food products using the Nutrition Information Panel (NIP) with one question displaying NIP with the same serving weights and the other with different serving weights. Of these, ten items were scored to give a total knowledge score. The remaining six items do not have a right or wrong answer and hence were not scored. Examples of these unscored questions include participant’s awareness of food labels, Na sources in their diet and if they compared nutrient content by per serving or per 100 g of the food item.

#### Behaviour

Twelve items were used to assess participants’ behaviour with regards to intakes of dietary Na. Of which, ten items were used to assess the frequencies (‘never’, ‘rarely’, ‘sometimes’, ‘often’, and ‘always’) of salt and Na use, and strategies employed to minimise Na intake (e.g. using spices or herbs instead of salt, minimising fast food consumption, ask to have less salt when eating out, etc). The remaining two items queried on participants’ current approach towards Na consumption (i.e., if participants are currently limiting their Na intake, the challenges faced if limiting Na intake, and reasons for not limiting Na intake).

#### Attitudes

Twenty-five items were used to assess participants’ attitudes toward dietary Na intake. These questions were assessed using a five-point Likert scale (e.g., ranging from ‘Strongly agree’ to ‘Strongly disagree’). Participants were asked to rate their concern of food-related issues (such as the amount of sugar, Na, and fat in food), their perceived ideas on salt alternatives such as monosodium glutamate (MSG) and potassium salts) and the availability of low Na dishes when eating out. Participants’ attitudes towards food labels related to Na and perceived responsibilities of different stakeholders on Na reduction were also queried.

#### Others

This section consists of six items. One matrix question with five items assessed participants’ interest in learning about dietary Na (e.g., learning more about the impact of Na on health, recommended Na intake, and ideas for reducing Na intake). These questions were assessed using a five-point Likert scale ranging from ‘Extremely interested’ to ‘Not at all interested’. The last item queried on their preferred sources for information on Na and health.

Demographic information was also collected and this included self-reported height, weight, existing medical conditions, participants’ household grocery shopping and cooking responsibilities, and if participants were recommended by others to reduce their Na intake. Using the self-reported height and weight, participants’ BMI was calculated, and they were categorised according to the WHO cut-off for Asian population^(31)^. Other demographic characteristics, such as age, sex, ethnicity, and education level were obtained from the Singapore Population Health Study Online Panel database which contains these socio-demographic data and updated yearly.

### Data analysis

Data were extracted from REDCap and imported to STATA/se Version 14·0 (StataCorp LP) for analysis. The data were weighted by gender, age, ethnicity, and education level to represent the Singapore resident population^(32)^. Descriptive statistics, mean and sd or *n* and proportion (%) were used to describe participant characteristics and responses. Agreement responses were aggregated into three categorical variables: ‘Strongly agree/Agree’, ‘Neither’, and ‘Strongly Disagree/Disagree’. Similarly, frequency responses to questions were aggregated into three categorical variables: ‘Never/Rarely’, ‘Sometimes’, and ‘Often/Always’.

To calculate a total knowledge score, scores from the ten knowledge questions were added up, with each question given a maximum score of one. For questions with multiple answers, each correct answer was given a score of one and the mean score was calculated. The minimum possible knowledge score is 0, while maximum is 10.

To calculate a total behaviour score, scores were awarded to the ten behavioural questions and added up. For each behavioural question, responses that indicated positive behaviours towards lower Na intake were given a score of one while a response with ‘Sometimes’ was given a score of 0·5. Responses indicative of negative behaviour (towards higher Na intake) were scored zero. The minimum possible behaviour score is 0, while maximum is 10.

*t* test and ANOVA were used to assess mean differences for knowledge and behaviour scores by socio-demographic subgroups. *χ* ^2^ tests were used to assess differences in attitudes related to dietary Na intake by socio-demographic subgroups. A *P* value of <0·05 was considered statistically significant.

## Results

A total of 955 participants completed the survey (77·0 % response rate). The mean age of the sample was 46·6 years (sd = 14·1), and there were slightly more females (58·0 %) than male (42·0 %) participants. A large proportion of the sample were Chinese (82·1 %), 10·5 % were Indian and 4·5 % were Malay. Almost half of the sample had attained a university degree or higher (47·3 %), 33·8 % completed education at pre-university level and 18·9 % attained secondary school education or lower. Just over half of the sample were overweight or obese (54·2 %), 14·9 % reported to be diagnosed with high blood pressure, and 13·2 % were on medication or making lifestyle changes due to high blood pressure. About one-eighth of the participants (11·9 %) had been recommended to reduce their dietary Na intake by a medical professional. About three-quarters of the participants reported that they had primary or shared responsibility for household grocery shopping (77·0 %), while about half of the participants were in charge or have shared responsibility for cooking in the household (49·4 %).

Compared with the Singapore population, people aged between 40 and 59 years, females and those of Chinese descent were overrepresented, while elderly aged 60 years and above, males and those of Malay descent were underrepresented. More details about the participant characteristics are shown in Appendix Table S1. In subsequent results below, the findings are based on weighted data.

### Declarative and procedural knowledge

The awareness of the health conditions related to high Na consumptions was relatively high among the participants (e.g., high blood pressure (91 %), kidney disease (85 %) and stroke (78 %)), and 59 % of the participants were able to correctly identify the relationship between salt and Na, (i.e., salt contains Na) (Appendix Table S2). However, a substantial proportion of the participants were also not aware of the recommended intakes for salt and Na intake (68 % and 83 %, respectively). Additionally, more than half of the participants had misconceptions about salt and dietary Na. For example, agreeing that Himalayan salt, pink salt, sea salt, and gourmet salts are healthier than regular table salt, Na intake can be reduced by replacing salt with chicken stock powder during cooking, and drinking more water can neutralise Na intake from their diet (See Appendix Table S2 for details). Majority of the participants (71–86 %) were able to identify common high Na foods (such as processed meats, fast foods and soya sauce), but only a smaller proportion of participants were able to identify foods with ‘hidden’ Na (such as processed seafood (53 %), ketchup (43 %), sliced cheese (41 %), chilli sauce (40 %), and corn flakes (7 %)) (see online supplementary material, Supplemental Fig. S1).

Regarding participants’ awareness of primary Na sources in their diets (Appendix Table S2), 34 % of the participants perceived that canned or processed foods contributed the most, while only 29 % and 13 % perceived that sauces and seasonings added during cooking and while eating contributed the most, respectively. When comparing their personal Na intake to their perceived recommended amount (Appendix Table S2), the proportions were quite evenly distributed among those who felt that they eat more, less, about the right amount and unsure of their Na intake.

For procedural knowledge (Appendix Table S3), while 58 % of the participants reported knowing how to monitor their Na intake based on the information on the NIP, only about a third of the participants (32 %) were able to correctly identify food items with higher Na content when the food labels presented different serving weights. Slightly over half of them (55 %) reported using the ‘per serving’ values rather than the ‘per 100 g’ values when making comparisons, suggesting misplaced knowledge in reading food labels.

When knowledge scores were related to demographics (Table 1), findings showed that participants with higher knowledge score tended to be in the younger age group (21–39 years), from the Chinese ethnic group, were not recommended by a medical professional to reduce their Na intake, had higher education attainment and BMI in the underweight/ideal weight category. Knowledge scores were not different between gender, among those with different medical history and treatment for high blood pressure and participants’ responsibilities in household grocery shopping and cooking.

**Table 1:** Knowledge and behaviour score in relation to participants’ characteristics (*n* 955)*

Characteristics	Knowledge score			Behaviour score		
	Mean	sd	*P*	Mean	sd	*P*
Age group†			**<0·01**			**<0·01**
21–39 years	4·7 ^a^	1·7		4·6 ^a^	1·9	
40–59 years	3·9 ^b^	1·6		5·4 ^b^	2·1	
≥ 60 years	4·2 ^b^	1·7		6·5 ^c^	1·9	
Gender			0·14			0·14
Male	4·1	1·7		5·4	2·2	
Female	4·3	1·7		5·5	2·0	
Ethnicity (*n* 927)†			**<0·01**			**<0·01**
Chinese	4·4 ^a^	1·7		5·4 ^a^	2·1	
Malay	3·5 ^b^	1·3		5·1 ^a^	2·1	
Indian	4·0 ^_^	1·7		6·0 ^b^	2·0	
Education†			**<0·01**			**<0·01**
Secondary school and below	3·5 ^a^	1·5		5·9 ^a^	1·9	
Post-secondary school	4·2 ^b^	1·7		5·4 ^b^	2·2	
University degree and above	4·9 ^c^	1·6		5·1 ^b^	2·0	
BMI categories (*n* 952)†			**<0·01**			**0·04**
Underweight/Ideal weight	4·4	1·7		5·6	2·1	
Overweight/Obese	4·1	1·7		5·3	2·1	
Medical history			0·09			**<0·01**
With high blood pressure	4·0	1·6		6·1	2·0	
Without high blood pressure	4·3	1·7		5·3	2·1	
Medical treatment for high blood pressure			0·19			**<0·01**
On medication or making lifestyle changes	4·0	1·6		6·2	2·0	
Not on medication or making lifestyle changes	4·3	1·7		5·3	2·1	
Recommended to reduce Na intake by medical professional			**0·01**			0·27
Yes	4·0	1·5		5·6	2·2	
No/ Do not know	4·3	1·7		5·4	2·1	
Responsibility in household grocery shopping			0·14			**<0·01**
In-charge or sharing responsibility	4·2	1·6		5·6	2·1	
Not responsible	4·4	1·8		4·8	2·1	
Responsibility in household cooking			0·64			**<0·01**
In-charge or sharing responsibility	4·3	1·7		5·8	2·0	
Not responsible	4·2	1·7		5·1	2·2	

^a,b,c^Mean values within a column with unlike superscript letters were significantly different (*P* < 0·05).

* *t* test on weighted data unless stated otherwise.

† ANOVA with Bonferroni correction on weighted data.

### Behaviours

Details of participants’ responses for the behavioural questions are summarised in Appendix Table S4. In brief, about half of the participants reported using salt, sauces or condiments frequently (i.e. often or always) when cooking (51 %), while only 13 % of them used these frequently at the table. Among the listed strategies to reduce Na consumption, the most common practice reported was minimising the consumption of preserved foods (51 %), followed by minimising fast foods, processed foods, savoury snacks (46 % to 41 %), and using spices or herbs instead of salt and condiments during cooking (31 %). Requesting for no or less salt when eating out was least frequently practiced (19 %) among the participants. A large proportion of the participants also reported noticing the NIP (94 %) and the Healthier Choice Symbol for ‘lower in Na’ (88 %) when doing grocery shopping (results not shown). However, only 28 % of the participants reported making use of food labels to check for Na content of food products and 32 % of them consumed low Na foods products frequently, although more of them reported doing this only sometimes (37 % and 38 %, respectively).

When related to demographics (Table 1), results showed that participants with higher behaviour scores tended to be those who are 60 years and older, from the Indian ethnic group, with history of high blood pressure, on medical treatment due to high blood pressure, had BMI in the underweight/ideal weight category, had primary or shared responsibility for household grocery shopping and cooking, and lower education attainment. There was no difference in scores between gender and those who were recommended by a medical professional to reduce Na intake.

In terms of current approaches towards Na consumption (Table 2), slightly more than half of the participants reported currently trying to limit or have tried to limit Na consumption, of whom most tended to be older, females, with secondary school education and below and have higher behaviour scores. Among these participants, a subset of them reported not knowing how to limit their Na intake and not having enough time to prepare food themselves. Those who reported these challenges are likely those who are younger, males and have higher education attainment. Only 4 % of the participants did not report any challenges faced. A key challenge reported by the participants was that the perception that food will not taste as good upon reduction of Na. This was also a common reason reported for those who are not limiting their Na intake (Table 2). Other reasons for not limiting Na intake includes being in good health, this is especially so for those with higher educational status, and not concerned about limiting Na, which is more commonly reported by males.

**Table 2:** Participants’ current approaches to limiting sodium consumption and barriers to limiting sodium intake

	Total %	Age				Gender			Ethnicity				Education				Knowledge score			Behaviour score		
		21–39 years %	40–59 years %	≥ 60 years %	*P*	Male %	Female %	*P*	Chinese %	Malay %	Indian %	*P*	Secondary school and below %	Post-secondary school %	University degree and above %	*P*	Mean	sd	*P*	Mean	sd	*P*
Which of the following best describes your approach to Na consumption? (*n* 955)†																						
Currently trying to Na /Have tried limiting Na	59·1	33·3	63·7	82·0	**	54·5	63·5	*	58·2	57·6	73·5		75·5	58·3	47·6	**	4·2	1·7		6·3	1·9	**
Not limiting Na	40·9	66·7	36·3	18·0		45·5	36·5		41·9	42·4	26·5		24·5	41·7	52·4		4·3	1·7		4·2	1·8	
Challenges faced when limiting Na intake (*n* 538)‡																						
Food does not taste as good if Na is reduced	59·4	55·7	60·2	60·3		55·6	62·5		59·9	61·2	66·3		65·9	56·3	56·5							
I do not know how to limit Na intake	22·3	23·4	26·2	17·0		28·7	17·2	**	22·1	19·8	20·4		22·1	19·8	20·4							
I do not have enough time to prepare food	19·4	38·8	17·5	12·7	**	16·7	21·6		20·3	14·5	18·8		11·7	21·7	25·3	*						
I lack the willpower to do so	9·3	12·4	10·3	6·6		11·7	7·4		10·7	1·9	11·1		8·9	8·3	11·4							
No challenges reported	4·1	3·2	0·4	9·1	**	3·4	4·7		1·8	19·0	4·1	**	7·2	3·2	1·6							
Reasons for not limiting Na intake (*n* 417)§																						
Food does not taste as good if Na is reduced	43·3	46·2	43·0	31·5		44·8	41·5		42·3	52·4	47·6		32·2	46·3	44·1							
I am in good overall health	36·8	39·8	30·5	44·0		35·0	38·9		38·7	27·7	40·5		23·1	30·3	48·5	**						
I do not know how to limit Na intake	32·7	39·9	26·3	22·1	*	35·2	29·7		32·3	37·8	25·3		34·7	34·1	30·5							
I do not really care that much	20·0	23·0	19·3	9·3		26·1	12·8	**	18·4	26·8	15·4		12·6	23·0	19·6							
I do not have enough time to prepare food	17·9	22·8	14·5	7·6		19·5	16·0		18·3	23·8	6·4		9·3	19·1	19·8							
I lack the willpower to do so	18·5	19·3	20·2	9·3		18·3	18·7		17·6	22·9	11·1		20·0	19·5	16·8							
I do not need as I am on blood pressure medication	4·6	3·9	4·6	8·0		6·7	2·2		1·5	19·9	6·4	**	3·2	8·6	1·0	**						

^a,b,c^Mean values within a column with unlike superscript letters were significantly different (*P* < 0·05).

* *P* < 0·05.

** *P* < 0·01.

† *n* 927 for Ethnicity, excluding responses from ‘Other’ ethnicity.

‡ *n* 524 for Ethnicity, excluding responses from ‘Other’ ethnicity.

§ *n* 403 for Ethnicity, excluding responses from ‘Other’ ethnicity.

### Attitudes towards dietary salt and sodium

A substantial proportion of the participants reported to be concerned about the amount of Na in foods and they tended to be those who are older, females and have higher behaviour scores (Table 3). Despite this, a large proportion of participants believed that salt is needed to make food tasty. Pertaining to the views on salt alternatives (Table 3), 64 % disagreed that MSG is a healthier alternative to table salt while 54 % were unsure if Na intake can be reduced by replacing table salt with potassium-enriched salts. Participants who were older and have higher knowledge and behaviour scores tended to disagree that MSG is a healthier alternative to table salt, while those who were younger and have lower behaviour scores were more likely to be unsure of using potassium-enriched salts to reduce Na intake.

**Table 3:** Attitudes towards sodium, salt and salt alternatives

Question†	Total %	Age				Gender			Ethnicity (*n* 927)				Education				Knowledge score			Behaviour score		
		21–39 years %	40–59 years %	≥ 60 years %	*P*	Male %	Female %	*P*	Chinese %	Malay %	Indian %	*P*	Secondary school and below %	Post-secondary school %	University degree and above %	*P*	Mean	sd	*P*	Mean	sd	*P*
How concerned are you about the amount of Na in food?																						
Concerned	78·3	66·5	77·7	93·1	**	72·9	83·3	**	79·9	72·1	84·4		80·1	78·8	76·2		4·3	1·7	*	5·8	2·0^a^	**
Neither	15·0	22·5	16·0	4·7		18·6	11·7		14·1	19·4	13·9		13·7	14·5	16·7		4·0	1·7 ^a^		4·1	1·7^b^	
Unconcerned	6·7	11·0	6·4	2·2		8·5	5·0		6·1	8·6	1·7		6·2	6·7	7·2		4·7	1·6^b^		3·6	1·6^b^	
I believe salt is needed to make food tasty.																						
Agree	73·3	74·9	76·5	66·4		74·9	71·8		73·1	76·4	82·9		68·3	73·7	76·8		4·2	1·6^a^	**	5·2	2·1^a^	**
Neither	13·8	13·5	13·6	14·4		14·0	13·6		13·9	15·3	11·6		15·3	14·6	11·6		3·9	1·7^a^		5·6	2·0^a^	
Disagree	12·9	11·6	10·0	19·2		11·1	14·6		13·0	8·3	5·5		16·5	11·7	11·7		5·0	1·9^b^		6·5	2·0^b^	
MSG is a healthier alternative to regular table salt.‡																						
Agree	8·8	5·9	9·5	10·7	*	7·1	10·5	**	8·0	15·2	4·4		14·0	9·2	4·3	**	3·7	1·7^a^	**	5·8	2·1^a^	**
Neither	27·4	37·0	22·9	24·9		33·7	21·3		28·1	29·5	16·1		21·2	32·6	25·1		4·0	1·7^a^		5·0	2·0^b^	
Disagree	63·8	57·1	67·7	64·3		59·2	68·2		64·0	55·3	79·5		64·8	58·2	70·6		4·4	1·7^b^		5·7	2·2^a^	
Na intake can be reduced by replacing table salt with potassium-enriched salts.‡																						
Agree	24·3	24·7	25·5	21·9	**	22·4	26·1		24·0	31·7	24·1		24·1	24·3	24·3		4·1	1·6		5·6	2·1	**
Neither	54·1	61·4	53·9	47·2		54·5	53·6		54·6	54·8	43·8		50·2	56·8	53·4		4·2	1·7		5·3	2·1^a^	
Disagree	21·7	14·0	20·6	31·0		23·1	20·3		21·4	13·5	32·1		25·7	18·9	22·4		4·5	1·9		6·1	2·1^b^	

^a,b,c^Mean values within a column with unlike superscript letters were significantly different (*P* < 0·05).

* *P* < 0·05.

** *P* < 0·01.

† Responses aggregated into three categories: ‘Concerned’ includes extremely and somewhat concerned, ‘Unconcerned’ includes not concerned and somewhat unconcerned, ‘Agree’ includes strongly and somewhat agree and ‘Disagree’ includes strongly and somewhat disagree.

‡ *n* 817 for all variables except Ethnicity (*n* 794) due to missing responses.

When eating out, majority of the participants felt that most foods available are high in Na or salt, with limited varieties of low Na options available (Table 4). With regards to manufactured food products, almost two-thirds of participants agreed that there should be laws to limit the amount of Na added to manufactured foods. About one-third of participants felt that is hard to find lower Na options for cooking and that lower Na options are more expensive, with a higher proportion of participants feeling neutral about both statements. In general, those who have neutral attitudes are likely to be reported by male participants and those with lower knowledge and behaviour scores.

**Table 4:** Views on sodium content in foods when eating out and low sodium food products

Question†	Total %	Age				Gender			Ethnicity (*n* 927)				Education				Knowledge score			Behaviour score		
		21–39 years %	40–59 years %	≥ 60 years %	*P*	Male %	Female %	*P*	Chinese %	Malay %	Indian %	*P*	Secondary school and below %	Post-secondary school %	University degree and above %	*P*	Mean	sd	*P*	Mean	sd	*P*
Most foods available when eating out (e.g. restaurants, food courts and cafes) are high in Na or salt.																						
Agree	83·4	81·2	80·8	90·0		82·4	84·3		87·0	65·1	83·6	**	80·6	81·0	88·5		4·3	1·7^a^	**	5·5	2·1	
Neither	12·9	14·9	15·9	5·9		13·6	12·3		9·9	27·9	14·6		16·0	15·3	7·6		3·4	1·7^b^		5·1	1·9	
Disagree	3·7	3·9	3·3	4·1		4·0	3·4		3·1	7·1	1·8		3·4	3·7	3·9		4·9	1·5^a^		5·1	1·9	
When eating out (e.g. restaurants, food courts and cafes), I find that lower Na options are not readily available or only in limited variety.																						
Agree	78·6	74·9	76·6	86·1		76·0	81·0		81·7	63·8	77·4	*	79·2	75·9	81·5		4·4	1·6^a^	**	5·6	2·2^a^	**
Neither	16·7	19·1	19·1	10·0		17·8	15·7		13·4	31·6	20·8		14·9	19·8	14·0		3·5	1·8^b^		4·9	1·8^b^	
Disagree	4·7	6·0	4·3	3·9		6·2	3·3		4·9	4·7	1·9		5·9	4·3	4·5		4·7	1·6^a^		5·4	2·0	
There should be laws which limit the amount of Na added to manufactured foods.																						
Agree	64·1	54·0	62·2	79·0	**	62·8	65·3		64·6	57·4	67·2		66·5	62·3	64·6		4·4	1·7^a^	**	5·8	2·1^a^	**
Neither	25·0	31·2	27·8	13·3		25·1	25·0		25·5	27·7	22·7		25·6	26·3	22·9		3·8	1·6^b^		5·0	2·0^b^	
Disagree	10·9	14·8	10·0	7·7		12·1	9·8		9·9	14·9	10·2		7·9	11·4	12·5		4·5	1·8^a^		4·5	1·8^b^	
It is hard to find lower Na options for cooking.‡																						
Agree	31·3	31·5	29·4	33·9		29·9	32·6	**	31·4	30·4	36·7		27·1	31·7	33·9		4·4	1·7^a^	**	5·5	2·2^a^	**
Neither	39·7	38·3	43·6	35·2		46·7	33·0		37·2	54·9	36·5		42·7	44·0	31·7		3·8	1·7^b^		5·2	2·0^a^	
Disagree	29·0	30·3	26·9	31·0		23·4	34·4		31·4	14·7	26·9		30·3	24·3	34·5		4·6	1·7^a^		6·1	2·1^b^	
Lower Na options are more expensive.‡																						
Agree	34·3	38·7	32·7	32·4		29·8	38·6	*	33·2	43·3	30·5		33·2	32·9	37·0		4·4	1·7		5·2	2·2^a^	**
Neither	43·4	40·7	48·0	39·0		43·2	43·6		42·7	41·9	50·2		43·9	43·7	42·5		4·1	1·7		5·6	2·0	
Disagree	22·4	20·7	19·4	28·7		27·0	17·9		24·1	14·9	19·3		23·0	23·4	20·5		4·4	1·7		5·9	2·3^b^	

^a,b,c^Mean values within a column with unlike superscript letters were significantly different (*P* < 0·05).

* *P* < 0·05.

** *P* < 0·01.

† Responses aggregated into three categories: ‘Agree’ includes strongly and somewhat agree, and ‘Disagree’ includes strongly and somewhat disagree.

‡ *n* 817 for all variables except ethnicity (*n* 794) due to missing responses.

Regarding the attitudes towards food labels (Table 5), those who reported to choose food products according to their experience or knowledge instead of referring to food labels tended to be those with lower behaviour scores. Although most participants reported to view a food or beverage more positively if it is advertised as lower in Na, about one-third of them felt confused about the usage of NIP and a small percentage of them do not trust the NIP. These attitudes are generally held by older, Malay participants, those with lower educational attainment, and lower knowledge scores.

**Table 5:** Attitudes towards food labels

Question†	Total %	Age				Gender			Ethnicity (*n* 927)				Education				Knowledge score			Behaviour score		
		21–39 years %	40–59 years %	≥ 60 years %	*P*	Male %	Female %	*P*	Chinese %	Malay %	Indian %	*P*	Secondary school and below %	Post-secondary school %	University degree and above %	*P*	Mean	sd	*P*	Mean	sd	*P*
I choose food products according to my experience or knowledge and not according to food labels																						
Agree	53·7	54·5	54·0	52·1		51·1	56·1		54·4	46·5	61·4		48·7	51·9	59·7		4·2	1·6^a^	**	5·2	2·1^a^	**
Neither	20·9	25·2	20·3	16·7		22·1	19·7		18·0	36·0	19·3		26·9	21·0	16·0		3·9	1·7^a^		5·3	1·8^a^	
Disagree	25·5	20·3	25·7	31·2		26·8	24·3		27·5	17·5	19·4		24·4	27·1	24·3		4·6	1·7^b^		6·1	2·1^b^	
I think more positively of a food or beverage product if it is advertised as ‘lower in salt/ Na’.																						
Agree	72·4	67·7	71·8	78·6		69·7	74·9		76·4	50·7	74·7	**	73·3	69·2	75·7		4·3	1·7^a^	**	5·6	2·1^a^	**
Neither	18·6	23·0	17·1	15n8		20·5	16·8		15·9	36·1	14·6		15·9	23·4	14·4		3·9	1·7^b^		4·8	2·1^b^	
Disagree	9·1	9·3	11·1	5·6		9·9	8·3		7·7	13·1	10·7		10·8	7·4	9·9		4·4	1·6		5·5	2·2	
I am confused about how to use the nutrition information panel to figure out how much Na is in the food I eat.																						
Agree	31·1	25·7	34·6	31·9	*	27·7	34·4		28·6	49·0	23·5	**	40·2	31·0	24·3	**	4·0	1·5^a^	**	5·3	2·2	
Neither	28·4	26·4	31·3	26·2		27·9	28·9		27·8	35·4	28·1		28·9	31·1	24·6		3·7	1·5^a^		5·4	2·0	
Disagree	40·5	47·9	34·2	41·9		44·4	36·8		43·6	15·5	48·4		30·9	38·0	51·1		4·8	1·8^b^		5·6	2·1	
I don’t trust the information labelled in the nutrition information panel																						
Agree	17·0	13·9	15·9	22·5	*	15·0	18·9		15·7	24·3	16·5	**	24·9	16·5	11·6	**	4·0	1·7^a^	**	5·8	2·3	
Neither	33·3	28·5	38·1	31·0		32·6	33·9		30·1	52·7	31·9		38·0	35·4	26·8		4·0	1·6^a^		5·4	2·1	
Disagree	49·8	57·6	46·0	46·5		52·4	47·2		54·1	23·0	51·6		37·2	48·2	61·5		4·5	1·7^b^		5·4	2·1	

^a,b,c^Mean values within a column with unlike superscript letters were significantly different (*P* < 0·05).

* *P* < 0·05.

** *P* < 0·01.

† Responses aggregated into three categories: ‘Agree’ includes strongly and somewhat agree, and ‘Disagree’ includes strongly and somewhat disagree.

When asked to rate the responsibility of various entities in reducing Na intake among Singapore residents (Appendix Fig. S2), participants rated themselves the highest (88 %). Other groups that are responsible include food manufacturers (78 %), friends/family (76 %), chefs (76 %) and fast-food chains (70 %). Government (65 %) and businesses (64 %) were rated lowest on the list.

### Others

**Fig. 1:**
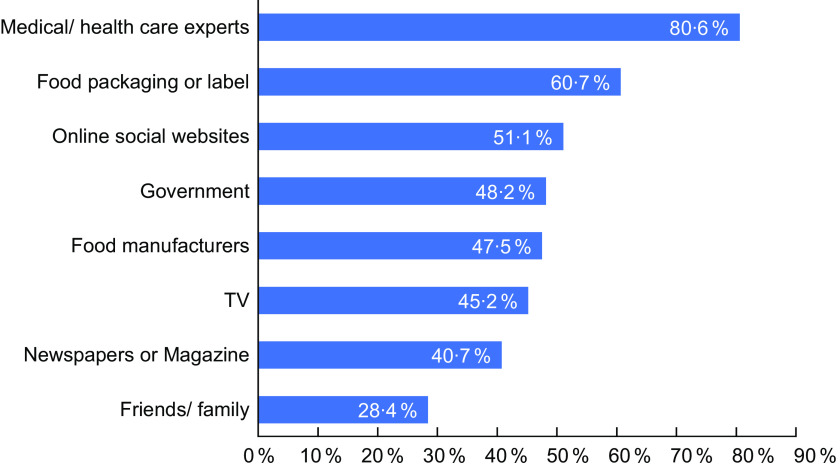
Preferred sources of information related to sodium

Pertaining to the topics related to dietary Na that participants are interested in learning (Appendix Fig. S3), participants were most interested in ideas for reducing Na, knowledge on the recommended Na intake and high Na food sources (80 % each). Most of the participants also reported to prefer information related to dietary intake to be disseminated by sources such medical or healthcare experts, food packaging or label and online social websites (Fig. 1) rather than newspapers or magazines and from family or friends.

## Discussion

This study demonstrated that while participants have good knowledge on the health effects of high Na consumption, their overall knowledge and behaviours scores related to dietary Na intake is comparatively low. These findings, together with the results of participants’ attitudes towards dietary Na and salt, could provide some insights to informing future Na awareness campaigns in Singapore.

Consistent with past studies among high income^(19)^ and Asian countries^(20,21,33)^, participants in this study were aware of the relationships between excess Na consumption and non-communicable diseases, such as high blood pressure and heart disease. Participants were also highly aware of the link between excess Na and kidney disease, which was less known in some countries, such as Australia and Barbados^(34,35)^. Furthermore, a large proportion of participants (59 %) were able to correctly identify the relationship between salt and Na, which was not the case in other studies (11–44 %)^(19)^. However, substantial knowledge gaps still exist among the population, such as knowing the recommended intakes of dietary salt and Na, and having misconceptions that specialty salts are healthier than table salt, that the use of chicken stock powder and drinking more water can reduce Na intake.

Another pertinent finding was that while a higher proportion of participants perceived that canned or processed foods contributed to most of the Na in their diets, there is evidence that most of Singapore population’s Na intake are from seasonings, salt and sauces (about 60 %), while only 37 % of dietary Na is contributed by processed foods^(12,36)^. Consistent with the previous observations in an international study^(37)^, it appears that participants may not be cognisant of the primary sources and the ubiquity of Na in their diet. This could explain why a substantial proportion of participants were unable to identify certain foods with ‘hidden’ Na and tended to underestimate their Na intake. Only 26 % believed that they are consuming more than the recommended amount while the 2018 National Nutrition Survey found that 90 % of Singaporeans exceeded the recommended amount of Na intake^(10)^. Taken together, there appears to be knowledge gaps on common and hidden sources of Na in the diet and a need to rectify misconceptions on products used for seasoning.

Our findings also highlighted that participants lacked the procedural knowledge to interpret Na content of food products when different serving weights were presented. Previous studies suggests that procedural knowledge is indicative of a person’s behaviour^(38)^. Thus, the low procedural knowledge among participants could explain the large proportion of them choosing food based on their experience and knowledge instead of using food labels to check for Na content. This is despite most reported noticing the NIP and Healthier Choice Symbol for lower in Na when shopping for food. Furthermore, our findings revealed that participants tend to wrongly perceive that they have the necessary knowledge to use foods labels to monitor their Na intake. Most were unable to read food labels correctly (69 %), even though 58 % reported knowing how to use food labels to monitor their Na intake. A small proportion of participants (30 %) also reported that they were confused with the usage of NIP. This hence warrants a need to better educate and equip the public with the skill to use these food labels effectively.

Similar to the results from past studies, our findings showed that those who are concerned about the Na content in food tended to exhibit more Na reduction behaviour^(28,30)^. However, only half of the participants reported currently limiting or have tried to limit their Na intake. The low proportion of participants practicing Na reduction behaviours can be attributed to many challenges as reported in our findings, and many of them could be averted through correcting certain misperceptions and increasing public awareness on practical skills^(37,39)^. For example, the misperception that taste will be reduced upon salt reduction and that Na reduction is not necessary for those in good health. Future Na reduction campaigns could investigate addressing these challenges to help improve the Na reduction behaviours among the population.

Another possible reason for the low behaviour scores could be due to the high frequency of eating out in Singapore^(40)^, where consumers are not able to have control over the amount of Na added to the dishes they purchase and consume. When asked about the Na content of foods out of home, most participants reported that these foods tended to be high in Na, and there were limited varieties of low Na option available. This highlights the need to work with food outlets in Singapore in controlling the Na content in out-of-home foods. While the current Healthier Dining Programme and Healthier Ingredient Schemes have increased the availability and accessibility of healthier food options in Singapore, expanding the guidelines in these programmes to include lower Na options may be helpful in reducing Na intake among the population. However, caution should be taken on whether such options should be explicitly endorsed on the menu as some consumers may develop negative taste expectations and compensate by adding more salt or sauces after the food is served^(41)^.

Besides limiting Na in the commonly consumed out-of-home foods, there could also be legislative actions to reduce Na content in manufactured food products, which many participants were supportive of, given that a substantial amount of Na is contributed by processed food in the population’s diet. Past studies have demonstrated the effectiveness of reformulation of food products^(42)^, with some suggesting adopting a covert approach (i.e. gradual reduction of Na without consumers noticing)^(41,43)^. Reformulation of manufactured products can also extend to sauces commonly used in Asian cuisine (e.g. soya sauce, oyster sauce and fish sauce), and this has been increasingly available in the market in the recent years^(44)^. However, it is worth noting that a substantial proportion of participants reported feeling neutral about the availability and cost of low Na products, which may suggest that they are not aware of the presence such food products on the market. Thus, more efforts should be done to increase the population’s awareness of these low Na products, as well as their understanding of salt alternatives like MSG and potassium-enriched salt, which have shown to help reduce dietary Na intake^(45,46)^. However, they were not well received among participants in this study, especially towards MSG, possibly due the negative press it received in the past^(47)^.

Past successful national Na reduction programmes in UK, Finland and Japan have demonstrated some key strategies that includes nutritional labelling, product reformulation and consumer awareness campaigns^(48–50)^. In Singapore, much effort has been devoted to the similar strategies like the Healthier Choice Symbol and Healthier Dining Programme^(13)^, but more must be done to increase public awareness. To inform the upcoming awareness campaign, it is crucial to understand what the population is interested to know and the communication channels to adopt for effective communication of health messages. Findings from this study highlighted that participant were interested to receive more information on the usage of food labels, ideas to reduce Na and the recommended intake amount and sources of high Na foods, through reliable sources. Furthermore, this study has helped identified sub-groups in the population that should be targeted for addressing the different aspects of KAB. For example, older participants and those with lower education attainment are more likely to have less knowledge, while younger participants and those with higher education attainment are more likely to have poorer Na-reduction behaviours.

To our knowledge, this is the first study exploring the KAB towards dietary Na among Singapore residents. Despite the large sample size and good response rates, there are several limitations in our study. First, although the data have been weighted, it must be noted that participants aged 60 years and above, males and Malay participants were underrepresented in the sample. As such, responses of these participants were up weighted and may limit the generalisation of our findings to the whole population. Second, as there was no appropriate validated tool relevant to the Singapore cultural and social context to measure Na related KAB, the questionnaire was self-developed by adapting from existing KAB questionnaires. Although it has been tested for face validity, it has not been tested for construct or criterion validity. Further research is warranted to validate this questionnaire for future use. Third, the data collected in the survey relied on self-reported responses and may be subjected to social desirability bias. We were also unable to quantify or validate the actual behaviours related to Na intake. Future research could consider using 24-h urine collection and multiple 24-h diet recalls to assess Na intake and its sources to complement this KAB questionnaire.

### Conclusion

This study has provided an overview of Na-related knowledge, attitudes and behaviours among residents in Singapore, contributing to the limited literature in this population. Substantial gaps in participants’ knowledge and skills have highlighted the need for more public education and improvements in the food environment (i.e., increase availability or low Na out-of-home foods and food products) to inform and empower Singapore residents in making healthier food choices.

## Supporting information

Chan et al. supplementary material 1 Chan et al. supplementary material

Chan et al. supplementary material 2 Chan et al. supplementary material

## Supplementary material

For supplementary material accompanying this paper visit https://doi.org/10.1017/S1368980023002422
