# Supplementary material for: Understanding knowledge, attitudes and behaviours related to dietary sodium intake in a multi-ethnic population in Singapore
Source: Public Health Nutr. 2023 Nov 3;26(12):2802–14. doi: 10.1017/S1368980023002422 (PMC10755448; doi:10.1017/S1368980023002422)
Supplement: Chan et al. supplementary material 1 — Chan et al. supplementary material [file S1368980023002422sup001.docx]

**Appendix S1.**

**SPHS Online Panel - Understanding knowledge, attitudes and behaviours related to dietary sodium intake in a multi-ethnic population in Singapore**

**Demographic information**

1. What is your height in cm? _________
2. What is your weight in kg? _________
3. Have you ever been diagnosed with or suffered from one or more of the following conditions? (Select all that apply)

- Asthma
- Cancer
- Diabetes
- High cholesterol
- Heart disease/ Heart attack
- High blood pressure
- Overweight/ Obesity
- Osteoporosis
- Stroke
- Others, please specify: __________________
- No diagnosed medical condition *(Skip Q4)*

1. Are you currently being treated for the condition(s)?

- Yes, currently taking medication
- Yes, making lifestyle changes only (e.g., changes to diet and physical activity)
- No, have not seen a doctor
- No, but have seen a doctor
- Don't know

1. Are you the main person who does the grocery shopping in your household?

- Yes
- No
- I share the responsibility

1. Are you the main person who does the cooking in your household?

- Yes
- No, someone else in the household does it
- No, we usually eat out or buy takeaways
- I share the responsibility

1. Has anyone ever recommended that you reduce your sodium intake?

- Yes *(Go to 68)*
- No *(Go to 70)*
- Don't know *(Go to 70)*

1. Who has recommended that you reduce your sodium intake? (Select all that apply)

- Medical professional
- Family member
- Friend
- Media source
- Others, please specify: ____________________

1. Why was it recommended that you reduce your sodium intake? (Select all that apply)

- To help treat/ avoid high blood pressure
- For overall health
- Prevent water retention
- To help treat/ avoid heart disease
- Eating a healthier diet now
- To help lose/ control weight
- Others, please specify: _______________________________________
- Do not know

**Declarative knowledge** (10 items)

1. When mentioned about "sodium", sometimes we hear about the term "salt" too. Which of these statements best describes the relationship between salt and sodium? *[Scores in brackets ()]*

- They are completely unrelated *(0)*
- They are exactly the same *(0)*
- Salt contains sodium *(1)*
- Sodium contains salt *(0)*
- Don't know *(0)*

1. Here is a list of food items, please select which food items you think are high in sodium content. (Select all that apply) *[Scores in brackets (), mean scores were calculated per participant]*

- Whitebread *(0)*
- Cake/ brownies/ muffins *(0)*
- Candy *(0)*
- Canned soup *(1)*
- Fast-foods (e.g., hamburger, pizza) *(1)*
- Grains/ pasta/ rice *(1)*
- Instant noodles with seasoning powder *(0)*
- Luncheon meat/ hot dogs/ sausages *(1)*
- Fish ball, fish cake, crab stick *(1)*
- Corn flakes *(1)*
- Savoury snacks (e.g., chips, crackers) *(1)*
- Sliced cheese *(1)*
- Oyster sauce *(1)*
- Soy sauce *(1)*
- Chilli sauce *(1)*
- Ketchup *(1)*

If you have answered in Question 6 that salt contains sodium, you are right!

Table salt is also called sodium chloride; hence it contains sodium.

Some examples of high sodium foods include: fast-foods, canned foods (e.g. luncheon meat, canned soups), processed foods (e.g. hot dog, sausages, fish ball, fish cake, instant noodles), savoury snacks (e.g. chips, crackers) and sauces (e.g. soy sauce, oyster sauce, chilli sauce, ketchup and gravies)

Please continue with the survey...

1. Of the following food items, where do you think does the SECOND MOST of the sodium in your diet come from? *[Question not scored]*

- Canned or processed foods (e.g., fish ball, fish cakes, luncheon meat, hotdogs)
- Naturally occurring sodium in foods
- Sodium from sauces and seasonings (e.g., salt, soy sauce, MSG, ketchup, chilli sauce) added while eating
- Sodium from sauces and seasonings (e.g., salt, soy sauce, MSG, ketchup, chilli sauce) added during cooking
- Restaurant/ take-away foods

1. What do you think is the maximum recommended amount of salt an average, healthy individual should consume in one day? *[Scores in brackets ()]*

- 10g (2 teaspoon) *(0)*
- 5g (1 teaspoon) *(1)*
- 2g (1/2 teaspoon) *(0)*
- Don't know *(0)*

1. What do you think is the maximum recommended amount of sodium an average, healthy individual should consume in one day? *[Scores in brackets ()]*

- 3000 mg *(0)*
- 2500 mg *(0)*
- 2000 mg *(1)*
- 1500 mg *(0)*
- Don't know *(0)*

1. How do you think your daily sodium intake compares to the recommended amount? *[Question not scored]*

- I eat more sodium than recommended
- I eat about the right amount of sodium
- I eat less than recommended
- Don't know

1. Which, if any, of the following conditions do you think is linked to eating too much sodium? Please make sure you select an option for each line. *[Scores in brackets (), mean scores were calculated per participant]*

|  |  | Yes | No | Do not know |
| --- | --- | --- | --- | --- |
| a. | High blood pressure | 🞏 *(1)* | 🞏 *(0)* | 🞏 *(0)* |
| b. | High blood sugar | 🞏 *(0)* | 🞏 *(1)* | 🞏 *(0)* |
| c. | Kidney disease | 🞏 *(1)* | 🞏 *(0)* | 🞏 *(0)* |
| d. | Heart disease/ heart attack | 🞏 *(1)* | 🞏 *(0)* | 🞏 *(0)* |
| e. | Stroke | 🞏 *(1)* | 🞏 *(0)* | 🞏 *(0)* |
| f. | Stomach cancer *[not scored]* | 🞏 | 🞏 | 🞏 |
| g. | Osteoporosis *[not scored]* | 🞏 | 🞏 | 🞏 |
| h. | Obesity *[not scored]* | 🞏 | 🞏 | 🞏 |

To what extent do you agree or disagree with the following statements? Please make sure you select an option for each line. *[Scores in brackets ()]*

|  |  | Strongly disagree | Somewhat disagree | Neither agree nor disagree | Somewhat agree | Strongly agree |
| --- | --- | --- | --- | --- | --- | --- |
|  | Himalayan salt, pink salt, sea salt and gourmet salts are healthier than regular table salt. | 🞏  *(1)* | 🞏  *(1)* | 🞏  *(0)* | 🞏  *(0)* | 🞏  *(0)* |
|  | Sodium intake can be reduced by replacing salt with chicken stock powder during cooking.  *(Chicken stock powder refers to dehydrated chicken broth, typically used as a flavour enhancer)* | 🞏  *(1)* | 🞏  *(1)* | 🞏  *(0)* | 🞏  *(0)* | 🞏  *(0)* |
|  | Drinking more water can neutralise sodium intake from my diet. | 🞏  *(1)* | 🞏  *(1)* | 🞏  *(0)* | 🞏  *(0)* | 🞏  *(0)* |

**Procedural knowledge** (6 items)

Food labels provide us with information about a product. The images below are two components of a food label.


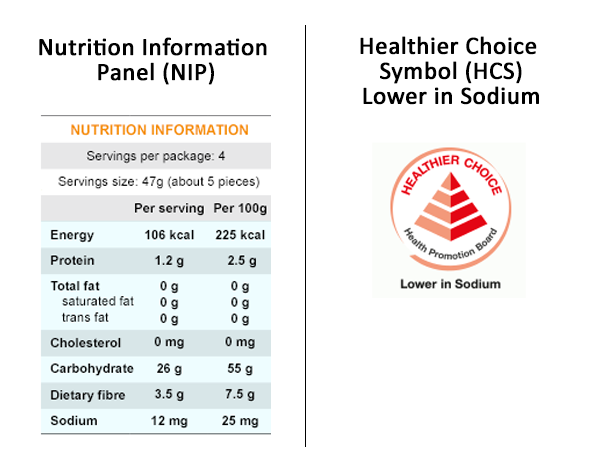


1. Have you ever seen the Nutrition Information Panel while shopping for food? *[Question not scored]*

- Yes
- No

1. Have you ever seen the Healthier Choice Symbol (Lower in sodium) while shopping for food? *[Question not scored]*

- Yes
- No

1. Here are the NIPs of three pasta sauces:


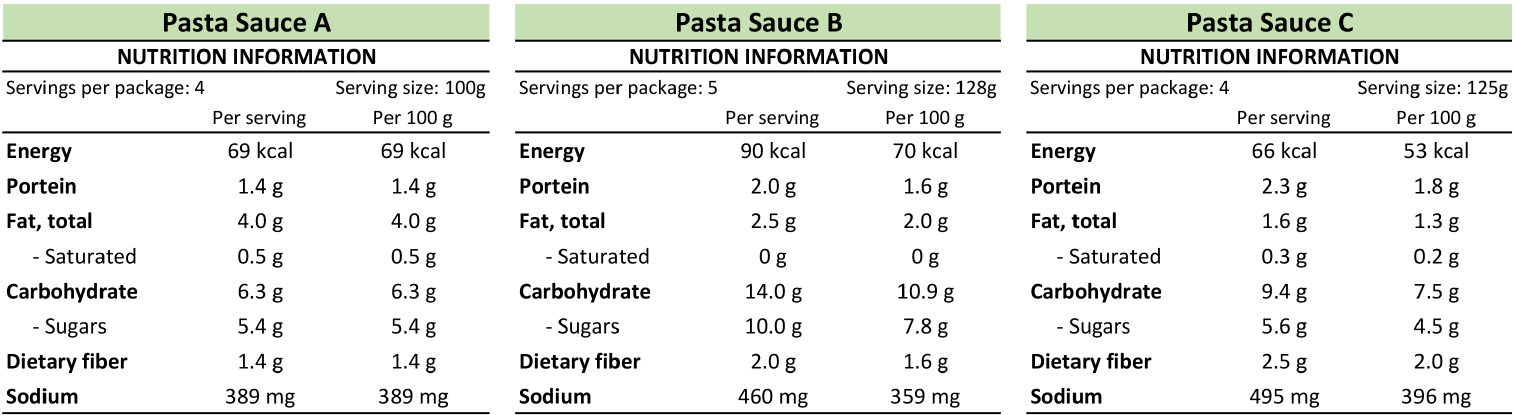


*[Note: different serving sizes presented]*

Which would you choose if you wish to minimise sodium intake? *[Scores in brackets ()]*

- Pasta sauce A *(0)*
- Pasta sauce B *(1)*
- Pasta sauce C *(0)*

1. Here are the NIPs of three tuna spreads:


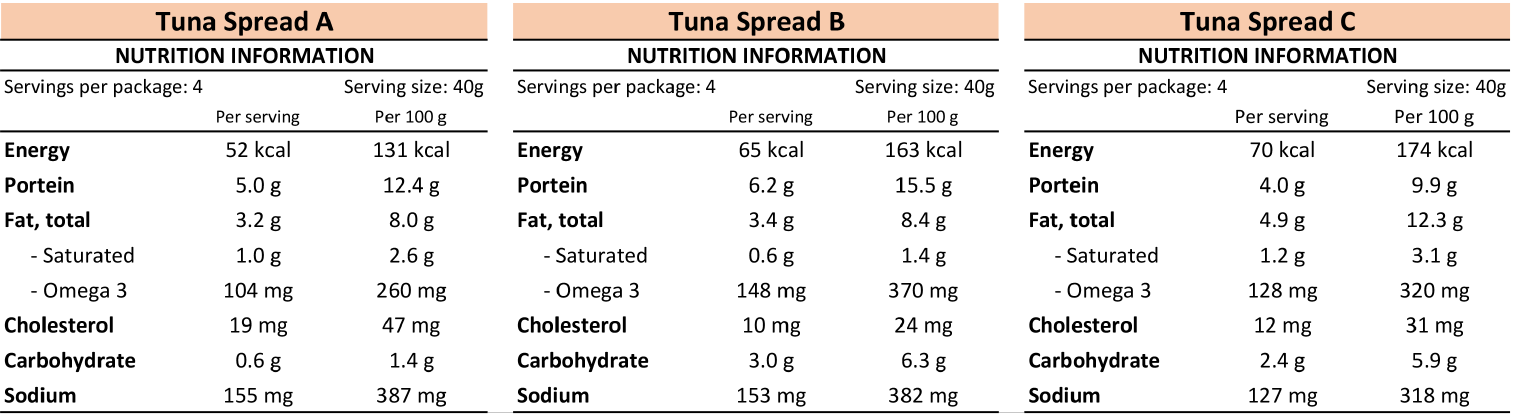


*[Note: same serving sizes presented]*

Which of the canned tuna spread has the highest sodium content? *[Scores in brackets ()]*

- Tuna spread A *(1)*
- Tuna spread B *(0)*
- Tuna spread C *(0)*

1. Here are the NIPs of two pasta sauces:


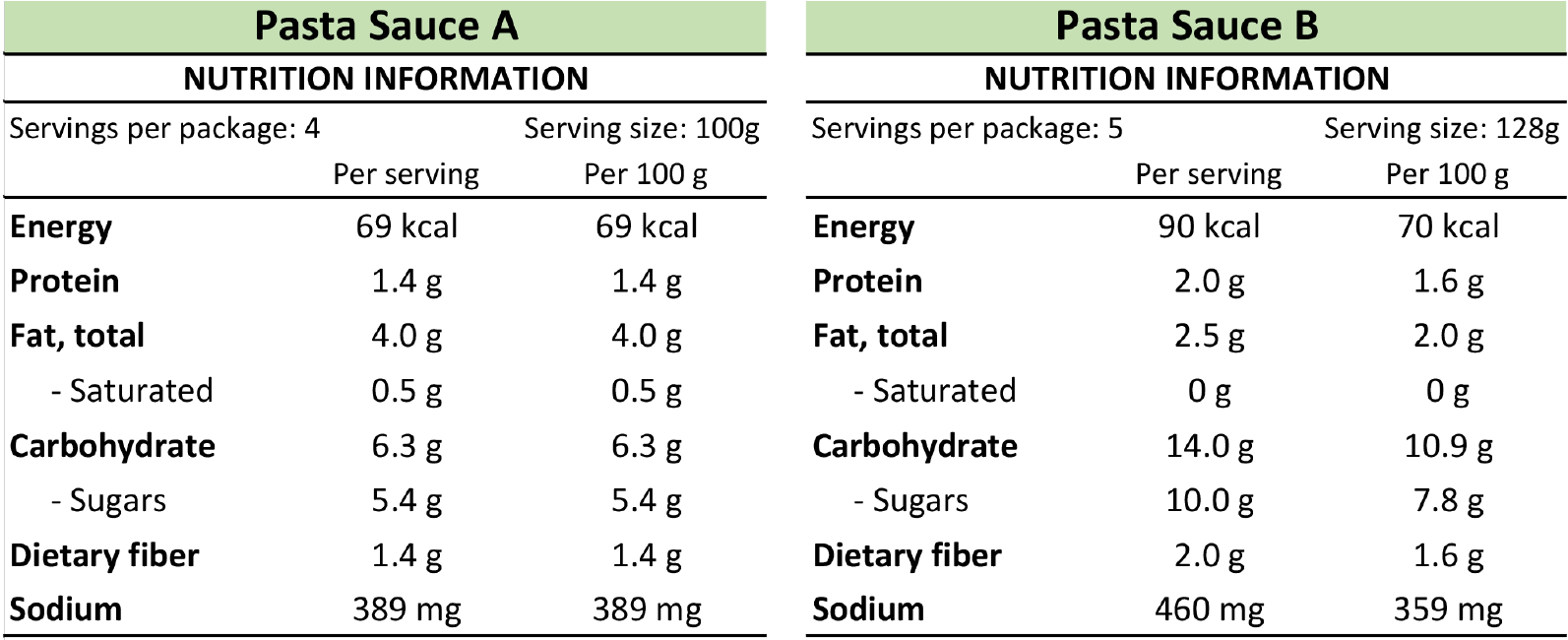


To choose a product with lower sodium content, would you look at the "per serving" values or "per values 100g" values? *[Question not scored]*

- "Per serving" values
- "Per 100g" values

1. I know how to monitor the amount of sodium I eat based on the information provided on the nutrition information panel. *[Question not scored]*
   - Strongly disagree
   - Somewhat disagree
   - Neither agree nor disagree
   - Somewhat agree
   - Strongly agree

**Behaviours related to sodium consumption** (12 items)

How often, if at all, do you do the following? Please make sure you select an option for each line. *[Scores in brackets ()]*

|  |  | Never | Rarely | Sometimes | Often | Always |
| --- | --- | --- | --- | --- | --- | --- |
|  | Add salt, sauce, or condiments (e.g., soy sauce, liquid seasoning, oyster sauce, chilli sauce) **at the table.** | 🞏  *(1)* | 🞏  *(1)* | 🞏  *(0.5)* | 🞏  *(0)* | 🞏  *(0)* |
|  | Add salt, sauce, or condiments (e.g., soy sauce, liquid seasoning, oyster sauce, chilli sauce) **when cooking.** | 🞏  *(1)* | 🞏  *(1)* | 🞏  *(0.5)* | 🞏  *(0)* | 🞏  *(0)* |
|  | Use spices/herbs instead of salt and condiments when cooking. | 🞏  *(0)* | 🞏  *(0)* | 🞏  *(0.5)* | 🞏  *(1)* | 🞏  *(1)* |
|  | Minimise consumption of snacks. | 🞏  *(0)* | 🞏  *(0)* | 🞏  *(0.5)* | 🞏  *(1)* | 🞏  *(1)* |
|  | Minimise consumption of pickles or preserved foods. | 🞏  *(0)* | 🞏  *(0)* | 🞏  *(0.5)* | 🞏  *(1)* | 🞏  *(1)* |
|  | Minimise consumption of pre-made/ ready-to-eat/ processed foods. | 🞏  *(0)* | 🞏  *(0)* | 🞏  *(0.5)* | 🞏  *(1)* | 🞏  *(1)* |
|  | Minimise eating food from fast-food restaurants (e.g., McDonalds, KFC, Pizza Hut). | 🞏  *(0)* | 🞏  *(0)* | 🞏  *(0.5)* | 🞏  *(1)* | 🞏  *(1)* |
|  | When eating out, asked to have your meal prepared with no or less salt and condiments. | 🞏  *(0)* | 🞏  *(0)* | 🞏  *(0.5)* | 🞏  *(1)* | 🞏  *(1)* |
|  | Looked at a food label to check the sodium content of a food item. | 🞏  *(0)* | 🞏  *(0)* | 🞏  *(0.5)* | 🞏  *(1)* | 🞏  *(1)* |
|  | Consumed foods labelled "sodium free", "low sodium" or "reduced sodium". | 🞏  *(0)* | 🞏  *(0)* | 🞏  *(0.5)* | 🞏  *(1)* | 🞏  *(1)* |

1. Which of the following best describes your approach to sodium consumption? *[Question not scored]*

- I do not need to limit sodium *(Go to 38b)*
- I am not interested in limiting sodium *(Go to 38b)*
- I am interested in limiting sodium, but I have not started to yet *(Go to 38a)*
- I have tried limiting sodium in the past, but I am not anymore *(Go to 38a)*
- I am currently trying to limit sodium *(Go to 38a)*

28a. What are some challenges you faced when controlling your sodium intake? (Select all that apply) *[Question not scored]*

- Food does not taste as good if sodium is reduced
- Lack of willpower
- Don't know/ lack information
- Don't have enough time to prepare food
- Others, please specify: __________________________________________

28b. Please share with us why are you not limiting your sodium intake? (Select all that apply) *[Question not scored]*

- I am in good overall health
- Food does not taste as good if sodium is reduced
- I don't really care that much
- Lack of willpower
- Don't know/ lack information
- Don't have enough time to prepare food
- I don't need to as I am on blood pressure medicine
- Others, please specify: __________________________________________

**Attitudes towards sodium and salt** (25 items)

To what extent do you agree or disagree with the following statements? Please make sure you select an option for each line.

|  |  | Strongly disagree | Somewhat disagree | Neither agree nor disagree | Somewhat agree | Strongly agree |
| --- | --- | --- | --- | --- | --- | --- |
|  | I believe salt is needed to make food tasty. | 🞏 | 🞏 | 🞏 | 🞏 | 🞏 |
|  | MSG is a healthier alternative to regular table salt. | 🞏 | 🞏 | 🞏 | 🞏 | 🞏 |
|  | Sodium intake can be reduced by replacing table salt with potassium-enriched salts | 🞏 | 🞏 | 🞏 | 🞏 | 🞏 |
|  | Most foods available out of home (e.g., from restaurants, food courts, cafes) are high in sodium or salt. | 🞏 | 🞏 | 🞏 | 🞏 | 🞏 |
|  | When eating out (e.g., at  Restaurants, food courts, cafes), I find that lower sodium options are not readily available or only in limited variety. | 🞏 | 🞏 | 🞏 | 🞏 | 🞏 |
|  | There should be laws which limit the amount of sodium added to manufactured foods. | 🞏 | 🞏 | 🞏 | 🞏 | 🞏 |
|  | It is hard to find lower sodium options for cooking. | 🞏 | 🞏 | 🞏 | 🞏 | 🞏 |
|  | Lower sodium options are more expensive. | 🞏 | 🞏 | 🞏 | 🞏 | 🞏 |

Please indicate on the scale below how concerned you are about each of the following

food-related issues? Please make sure you select an option for each line.

|  |  | Not at all concerned | Somewhat concerned | Neither concerned nor unconcerned | Somewhat concerned | Extremely concerned |
| --- | --- | --- | --- | --- | --- | --- |
|  | Healthy eating | 🞏 | 🞏 | 🞏 | 🞏 | 🞏 |
|  | The amount of sugar in food | 🞏 | 🞏 | 🞏 | 🞏 | 🞏 |
|  | The amount of sodium in food | 🞏 | 🞏 | 🞏 | 🞏 | 🞏 |
|  | The amount of fat in food | 🞏 | 🞏 | 🞏 | 🞏 | 🞏 |
|  | The amount of cholesterol in food | 🞏 | 🞏 | 🞏 | 🞏 | 🞏 |
|  | The amount of calories in food | 🞏 | 🞏 | 🞏 | 🞏 | 🞏 |

To what extent do you agree or disagree with the following statements regarding food labels (e.g., HCS and NIP)? Please make sure you select an option for each line.

|  |  | Strongly disagree | Somewhat disagree | Neither agree nor disagree | Somewhat agree | Strongly agree |
| --- | --- | --- | --- | --- | --- | --- |
|  | I choose food products according to experience or knowledge and not according to food labels. | 🞏 | 🞏 | 🞏 | 🞏 | 🞏 |
|  | I think more positively of a food or beverage product if it is advertised as "lower in salt/sodium". | 🞏 | 🞏 | 🞏 | 🞏 | 🞏 |
|  | I am confused about how to use the Nutrition information panel to figure out how much sodium is in the food I eat. | 🞏 | 🞏 | 🞏 | 🞏 | 🞏 |
|  | I don't trust the information labelled in the nutrition information panel. | 🞏 | 🞏 | 🞏 | 🞏 | 🞏 |

From the list below please rate how responsible you think each group is for reducing the amount of sodium Singaporeans eat. Please make sure you select an option for each line.

|  |  | Not at all responsible | Somewhat not responsible | Neutral | Somewhat responsible | Very responsible | Do not know |
| --- | --- | --- | --- | --- | --- | --- | --- |
|  | Government | 🞏 | 🞏 | 🞏 | 🞏 | 🞏 | 🞏 |
|  | Food manufacturers | 🞏 | 🞏 | 🞏 | 🞏 | 🞏 | 🞏 |
|  | Business  (e.g., supermarkets, local markets) | 🞏 | 🞏 | 🞏 | 🞏 | 🞏 | 🞏 |
|  | Chefs preparing foods in restaurants/ food courts/ cafes | 🞏 | 🞏 | 🞏 | 🞏 | 🞏 | 🞏 |
|  | Family / friends | 🞏 | 🞏 | 🞏 | 🞏 | 🞏 | 🞏 |
|  | Yourself | 🞏 | 🞏 | 🞏 | 🞏 | 🞏 | 🞏 |
|  | Fast-food chains | 🞏 | 🞏 | 🞏 | 🞏 | 🞏 | 🞏 |

**Others** (5 items)

How interested, if at all, would you be in learning the following? Please make sure you select an option for each line.

|  |  | Not at all interested | Somewhat uninterested | Neither interested not uninterested | Somewhat interested | Extremely interested |
| --- | --- | --- | --- | --- | --- | --- |
|  | Learning more about the impact of sodium on health. | 🞏 | 🞏 | 🞏 | 🞏 | 🞏 |
|  | Foods and beverages that contain the highest amounts of sodium per serving. | 🞏 | 🞏 | 🞏 | 🞏 | 🞏 |
|  | The amount of sodium I should be consuming. | 🞏 | 🞏 | 🞏 | 🞏 | 🞏 |
|  | What types of people should be concerned with sodium intake. | 🞏 | 🞏 | 🞏 | 🞏 | 🞏 |
|  | Ideas for reducing sodium intake. | 🞏 | 🞏 | 🞏 | 🞏 | 🞏 |

1. From which of the following sources would you want to hear about sodium and its impact on health? (Select all that apply)

- Medical/ health care experts
- Food packaging or label
- Government
- Food manufacturers
- Magazines
- TV
- Newspapers
- Friends/ family
- Online social websites
- Others, please specify: ________________________________________
- Don't know
