# Supplementary material for: Understanding knowledge, attitudes and behaviours related to dietary sodium intake in a multi-ethnic population in Singapore
Source: Public Health Nutr. 2023 Nov 3;26(12):2802–14. doi: 10.1017/S1368980023002422 (PMC10755448; doi:10.1017/S1368980023002422)
Supplement: Chan et al. supplementary material 2 — Chan et al. supplementary material [file S1368980023002422sup002.docx]

# Appendix Tables and Figures

| **Table S1. Characteristics of participants (n = 955)** | | | |
| --- | --- | --- | --- |
| **Characteristic** | **Sample population  % (n)** | **Weighted sample population %** | **Singapore population % †** |
| Age (mean ± SD) | 46.6 ± 14.1 | 48.0 ± 14.5 |  |
| Age group |  |  |  |
| 21 - 39 y | 34.9 (333) | 31.0 | 33.9 |
| 40 - 59 y | 42.7 (408) | 42.4 | 38.0 |
| 60 y and older | 22.4 (214) | 26.1 | 28.2 |
| Gender |  |  |  |
| Male | 42.0 (401) | 48.0 | 48.3 |
| Female | 58.0 (554) | 52.0 | 51.7 |
| Ethnicity ‡ |  |  |  |
| Chinese | 82.1 (784) | 76.0 | 76.1 |
| Malay | 4.5 (43) | 12.7 | 12.4 |
| Indian | 10.5 (100) | 8.4 | 8.5 |
| Others | 2.9 (28) | 3.0 | 3.0 |
| Education |  |  |  |
| Secondary school and below | 18.9 (180) | 25.1 | 36.9 |
| Pre-University/ Post-secondary | 33.8 (323) | 42.3 | 26.9 |
| University degree or higher | 47.3 (452) | 32.6 | 36.2 |
| BMI (mean ± SD) § | 24.4 ± 4.8 | 24.6 ± 5.0 |  |
| BMI Categories § |  |  |  |
| Underweight / Ideal weight | 45.8 (436) | 43.9 |  |
| Overweight / Obese | 54.2 (516) | 56.1 |  |
| Diagnosed with high blood pressure | 14.9 (142) | 16.5 |  |
| On medication or making lifestyle changes for high blood pressure | 13.2 (126) | 15.1 |  |
| Recommended to reduce sodium intake by medical professional |  |  |  |
| Yes | 11.9 (114) | 15.4 |  |
| No/ do not know | 88.1 (841) | 84.6 |  |
| In-charge of grocery shopping in the household |  |  |  |
| Yes, or shared | 77.0 (735) | 76.6 |  |
| No | 23.0 (220) | 23.4 |  |
| In-charge of cooking in the household |  |  |  |
| Yes, or shared | 49.4 (472) | 46.4 |  |
| No | 50.6 (483) | 53.6 |  |
| † Data taken from Department of Statistics, Ministry of Trade and Industry, Singapore ^(1)^  ‡ Participants of the “Others” ethnic group were excluded from subsequent analysis due to the small sample size  § *n* = 952 as *n* = 3 has missing height or weight values. BMI was calculated using kg/m^2^ and categorized using the WHO cut-off for the Asian population ^(2)^ | | | |

| **Table S2. Declarative knowledge on dietary sodium and salt** (*n* = 955) | | |
| --- | --- | --- |
|  | **Weighted %** | **95% Confidence interval** |
| ***Questions used for knowledge scores*** |  |  |
| Knows that high sodium intake is: |  |  |
| Associated with high blood pressure | 90.9 | 88.5 - 92.8 |
| Associated with kidney disease | 84.9 | 81.7 - 87.7 |
| Associated with stroke | 78.0 | 74.8 - 80.9 |
| Associated with heart disease | 74.3 | 70.9 - 77.4 |
| Not associated with high blood sugar | 61.5 | 57.6 – 65.2 |
| Knows that salt contains sodium | 59.1 | 55.3 - 62.9 |
| Knows the recommended daily salt intake | 32.1 | 28.6 - 35.8 |
| Knows the recommended daily sodium intake | 16.8 | 14.2 - 19.9 |
| Knows that specialty salts are not healthier than table salt | 10.0 | 8.2 - 12.1 |
| Knows that sodium intake cannot be reduced by using chicken stock powder | 38.8 | 35.3 - 42.5 |
| Knows that drinking water does not neutralise sodium intake from diet | 23.6 | 20.7 - 26.8 |
| No. of high sodium sources identified (*n* = 13 items in total): |  |  |
| 0 to 3 | 8.5 | 6.5 - 11.1 |
| 4 to 8 | 42.7 | 38.9 - 46.6 |
| 9 to 13 | 48.7 | 44.9 - 52.6 |
|  |  |  |
| ***Exploratory questions*** |  |  |
| Perceived main source of sodium in diet |  |  |
| Canned or processed foods (e.g., fish ball, fish cakes, luncheon meat,   hotdogs) | 34.5 | 30.9 - 38.2 |
| Sodium from sauces and seasonings (e.g., salt, soy sauce, MSG,   ketchup, chilli sauce) added during cooking | 28.5 | 25.2 - 31.9 |
| Restaurant/ take-away foods | 20.2 | 17.4 - 23.3 |
| Sodium from sauces and seasonings (e.g., salt, soy sauce, MSG,   ketchup, chilli sauce) added while eating | 12.6 | 10.0 - 15.8 |
| Naturally occurring sodium in foods | 4.3 | 3.0 - 6.2 |
| Personal sodium intake in comparison to recommended amount |  |  |
| I eat more sodium than recommended | 25.7 | 22.5 - 29.1 |
| I eat about the right amount of sodium | 28.0 | 24.8 - 31.5 |
| I eat less than recommended | 21.9 | 18.9 - 25.4 |
| I do not know | 24.4 | 21.1 - 28.0 |

| **Table S3. Procedural knowledge on dietary sodium and salt** (*n* = 955) | | |
| --- | --- | --- |
|  | **Weighted %** | **95% Confidence interval** |
| ***Questions used for knowledge scores*** |  |  |
| Knows how to use the Nutrient Information Panel to select food products when: | | |
| Different serving sizes presented | 31.6 | 28.3 - 35.1 |
| Same serving sizes presented | 71.7 | 67.9 - 75.2 |
|  |  |  |
| ***Exploratory questions*** |  |  |
| Have seen the Nutrition Information Panel while shopping for food | | |
| Yes | 94.3 | 92.1 - 95.9 |
| No | 5.7 | 4.1 - 7.9 |
| Have seen the Healthier Choice Symbol (Lower in Sodium) while shopping for food | | |
| Yes | 88.4 | 85.6 - 90.8 |
| No | 11.6 | 9.2 - 14.4 |
| Reference column when comparing sodium content (*n* = 817) |  |  |
| "Per serving" values | 55.2 | 51.1 - 59.2 |
| "Per 100 g" values | 44.8 | 40.8 - 48.9 |
| I know how to monitor the amount of sodium I eat based on the information provided on the nutrition information panel. | | |
| Agree | 57.6 | 53.8 - 61.5 |
| Neither | 23.7 | 20.4 - 27.1 |
| Disagree | 18.8 | 15.7 - 22.0 |

**Figure S1. Percentage of participants who identified these foods as high in sodium**

| **Table S4. Behaviour towards dietary sodium and salt** | | | |
| --- | --- | --- | --- |
| **Behaviour** | % (95% Confidence interval) | | |
|  | **Never/ Rarely** | **Sometimes** | **Often/ Always** |
| *Salt and sodium use* |  |  |  |
| Use salt, sauces, or condiments when cooking | 13.0 (10.6 - 15.8) | 36.2 (32.3 - 40.2) | 50.9 (47.0 - 54.7) |
| Use salt, sauces, or condiments at the table | 46.3 (42.5 - 50.1) | 41.6 (37.8 - 45.4) | 12.1 (10.0 - 14.6) |
| *Strategies to reduce sodium consumption* |  |  |  |
| Minimise consuming pickled or preserved foods (e.g., salted vegetables, achar) | 12.2 (9.7 - 15.3) | 37.4 (33.8 - 41.2) | 50.5 (46.7 - 54.4) |
| Minimise consuming fast foods (e.g., burgers, fried chicken, pizza) | 17.0 (14.2 - 20.2) | 37.2 (33.6 - 41) | 46.0 (42.2 - 49.8) |
| Minimise consuming ready-to-eat or processed foods (e.g., luncheon meat, sausages, fish balls) | 16.2 (13.5 - 19.4) | 38.4 (34.8 - 42.1) | 45.5 (41.7 - 49.4) |
| Minimise consuming savoury snacks (e.g., chips and crackers) | 15.0 (12.3 - 18.1) | 44.0 (40.3 - 47.9) | 41.1 (37.4 - 44.9) |
| Use spices or herbs instead of salt and condiments when cooking | 24.3 (21.3 - 27.6) | 44.9 (41.1 - 48.8) | 30.9 (27.6 - 34.4) |
| Consume foods products labelled "sodium free", "low sodium" or "reduced sodium" | 29.8 (26.5 - 33.4) | 38.4 (34.7 - 42.2) | 31.9 (28.5 - 35.6) |
| Look at the food labels or Nutrition Information Panel to check the sodium content of a food item | 35.6 (32.1 - 39.3) | 36.4 (32.8 - 40.2) | 28.1 (24.8 - 31.7) |
| When eating out, request to have your meal prepared with no or less salt and condiments | 50.6 (46.8 - 54.4) | 30.5 (27 - 34.2) | 19.0 (16.2 - 22.2) |

**Figure S2. Responsibility of various entities in reducing sodium intake among Singapore residents**

**Figure S3. Topics of interest related to dietary sodium**

**References**

1. Department of Statistics Singapore (2021) Singapore Census of Population 2020, Statistical Release 1: Demographic Characteristics, Education, Language and Religion. <https://www.singstat.gov.sg/publications/reference/cop2020/cop2020-sr1/census20_stat_release1> (accessed 7 October 2021)

2. WHO Expert Consultation (2004) Appropriate body-mass index for Asian populations and its implications for policy and intervention strategies. *Lancet (London, England)* 363, 157-163.
